# Neuropsychiatric and Neurological Diseases in Relation to the Microbiota-Gut-Brain Axis: From Research to Clinical Care

**DOI:** 10.7759/cureus.44819

**Published:** 2023-09-07

**Authors:** Purva Gulrandhe, Sourya Acharya, Samarth Shukla, Maharshi Patel

**Affiliations:** 1 Department of Physiotherapy, Ravi Nair Physiotherapy College, Datta Meghe Institute of Higher Education and Research, Wardha, IND; 2 Department of Medicine, Jawaharlal Nehru Medical College, Datta Meghe Institute of Higher Education and Research, Wardha, IND; 3 Department of Pathology, Jawaharlal Nehru Medical College, Datta Meghe Institute of Higher Education and Research, Wardha, IND

**Keywords:** central nervous system, neurology, microbiota, neurological disorders, gut-brain axis

## Abstract

Neurological disease is on the upswing, the second leading cause of mortality and a significant cause of disability. The term *gut-brain axis* emphasizes a dynamic two-way communication between the central nervous system and the gastrointestinal system. The microbiome is being linked to more and more clinical and preclinical studies as a major risk factor for neurological diseases. Overall, 288 studies were identified initially. After screening, data extraction, and applying the inclusion and exclusion criteria, 37 articles were included in the study. Changes in the gut microbial population composition have been correlated to many neurological and neurodevelopmental diseases.

## Introduction and background

Globally, neurological diseases are on the rise, making them the second leading cause of mortality and the primary reason for disability. The burden of disability and death related to neurological diseases is increasingly recognized as a worldwide public health concern, and this burden is predicted to increase in the coming decades [1]. The concept of the *gut-brain axis* underscores a dynamic and reciprocal interaction between the gastrointestinal (GI) system and the central nervous system (CNS). Within this interplay, the CNS typically governs the activities and operations of the GI tract. Furthermore, the hypothalamic-pituitary-adrenal (HPA) axis, responsible for regulating stress responses, plays a role in influencing gut function through its modulation of hormonal factors.

On the other hand, it is hypothesized that the GI system may influence the CNS, affecting various aspects of the brain, including behavior, cognition, and nociception [2]. Through chemical communication, including *indirect* and *direct* signaling, the gut microbiota can influence homeostasis and behavior in its animal host. The gut microbiota also affects neurotransmitter (NT) concentrations in model systems, suggesting that microbes act as mediators of typical signaling molecules used by the nervous system. Additionally, the gut and the brain are physically linked via neural connections, with the vagus nerve being the most important of these neural pathways. Gut bacteria strongly influence the growth and functioning of the peripheral immune system, and the microbiota is essential for the proper maturation, activation, and growth of microglia, which are innate immune cells in the brain [3]. Increasingly, the microbiome is being linked to clinical and preclinical studies as a potentially major risk factor for neurological diseases such as multiple sclerosis (MS), autism spectrum disorder (ASD), Parkinson's disease (PD), Alzheimer's disease (AD), and stroke [4].

## Review

Methodology

Search Strategy

**Figure 1:**
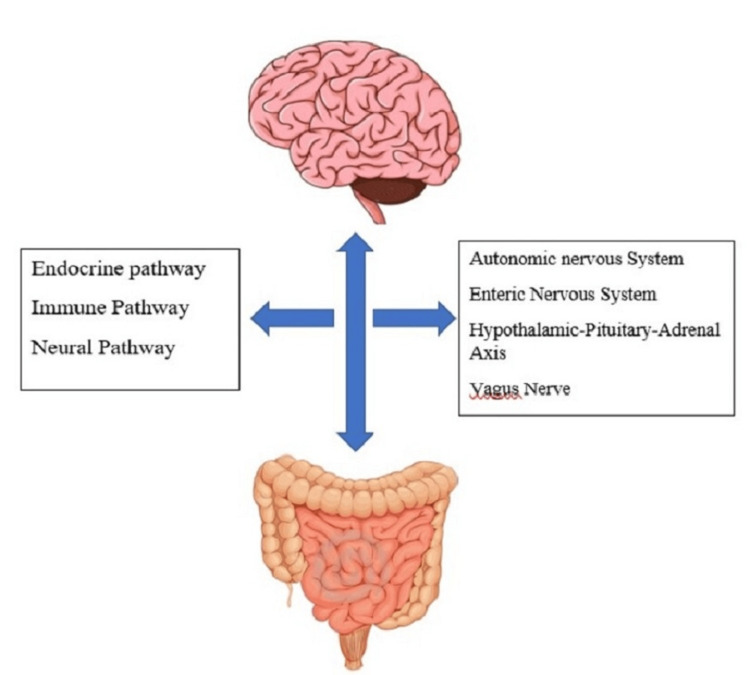
Gut-brain relationship through various connections. Image credit: Maharshi Patel.

**Figure 2:**
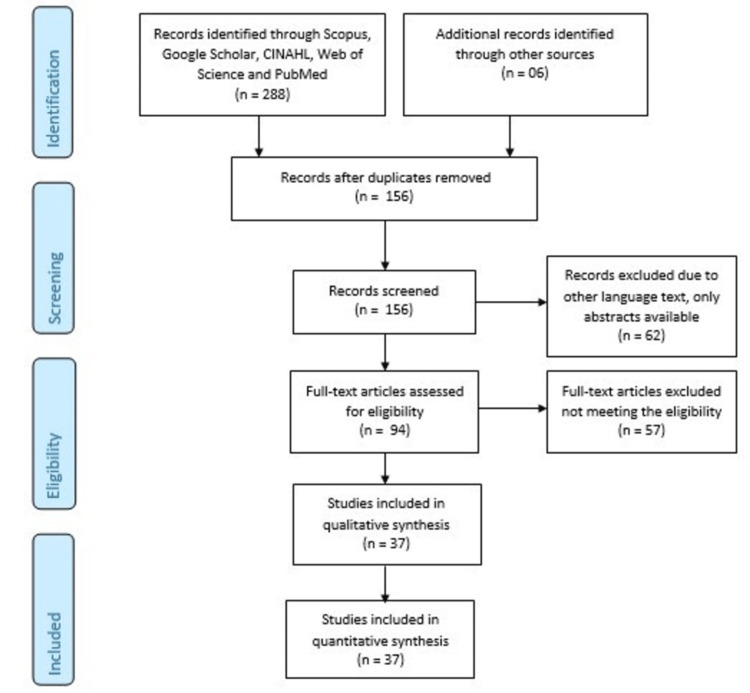
A summary of the selected articles has been provided in accordance with PRISMA guidelines. PRISMA, Preferred Reporting Items for Systematic Reviews and Meta-Analyses; CINAHL, Cumulative Index to Nursing and Allied Health Literature

Between 2013 and 2023, we conducted an electronic search in Scopus, Google Scholar, Cumulative Index to Nursing and Allied Health Literature (CINAHL), Web of Science, and PubMed. The search terms used were "central nervous system," "neurological disorders," and "brain-gut axis." We employed Boolean terms such as "AND," "OR," and "WITH" in the same search. The initial number of articles searched was 294, and after screening, 110 were selected. Following an overall screening process, the number of articles included in the study was 37. Figure 1 illustrates the bidirectional relationship between the gut and the brain. A summary of the selected articles can be found in the Preferred Reporting Items for Systematic Reviews and Meta-Analyses (PRISMA) flowchart (Figure 2).

Data Extraction

Each article was separately analyzed by two review authors who abstracted primary and secondary data for data collection. The search yielded numerous papers, including editorials, abstracts, review articles, and free full texts. After a thorough analysis of these papers, a search for additional publications was conducted using applicable articles and their references. The following criteria were used to select these review papers: observational research, publications in the English language, studies published between 2013 and 2023, and review articles.

Results 

A total of 288 studies were identified, and the step-by-step procedure for article selection is detailed in the PRISMA flowchart. The electronic databases used included PubMed, CINAHL, Google Scholar, and Scopus. Initially, 156 records were reviewed during the screening process, leading to the exclusion of 138 articles. The availability of a full-text copy determined the eligibility of the articles. Eventually, 94 articles met the eligibility requirements, but 57 of them were excluded due to errors, duplicates, and other irregularities. The study comprised a total of 37 selected articles, which focused on neurological disorders such as mood and anxiety issues, MS, PD, AD, migraines, autism spectrum disorder (ASD), schizophrenia, as well as stroke.

Gut-brain axis and neurologic diseases

Multiple Sclerosis

The gut microbiota potentially influences the pathology of MS. Individuals with relapsing-remitting MS have an abundance of Mycoplasma, Pseudomonas, Faecalibacterium, Haemophilus, Anaerostipes, Blautia, and Dorea, while Bacteroides, Prevotella, Parabacteroides, and Adlercreutzia are relatively less common. Children with MS exhibit low levels of Lachnospiraceae and Ruminococcaceae and significant levels of Desulfovibrionaceae members [5]. NTs have roles in both the peripheral nervous system and the CNS, and they are produced by neurosecretory or neuroendocrine cells. Intestinal bacterial species affect the number of host metabolites required for generating NTs in the CNS. Furthermore, certain gut flora secretes various NTs, such as 5-hydroxytryptamine (5-HT), gamma-aminobutyric acid (GABA), and dopamine, controlling their peripheral levels. These NTs also interact with specific immune cell receptors, influencing the immune system. In MS, NTs act as potent mediators of gut-brain interactions, with 5-HT possibly playing a dominant role due to its connection to neuroinflammatory diseases, its impact on the neurological and immune systems, and its microbial regulation [6]. Evidence supports the cytokine hypotheses of MS-related fatigue and depression, indicating that immune-inflammatory pathways contribute to the development of depression and fatigue in MS [7].

In patients, a specific group of CD4+ helper T cells undergoes a reduction in quantity and shifts from a regulatory to a pro-inflammatory role. These cells are drawn to the gut-associated lymphoid tissue, an immunological organ closely interacting with the intestinal microbiota. This phenomenon suggests an immune regulatory influence in secondary progressive MS (SPMS), a disease stage characterized by debated immunological origins. These gut-homing T cells act as biological messengers, potentially conveying altered signaling in the brain [8].

Mood and Anxiety Problems

Mental health disorders are expected to emerge as a pressing crisis in the coming times [9]. Mood disorders are significantly influenced by the gut-brain axis, and researchers have proposed three probable mechanisms: (1) a strong infection initially triggers the immune system, ultimately resulting in mania; (2) mania patients with high bacterial infections show a low response condition in the immune system; and (2) antibiotic use may alter the microbiota, increasing the likelihood of changes in mood states [10]. Both acute stress and chronic stress have various impacts on the intestinal microbiota. Acute stress has a negligible effect due to the microbiota's long-term relative stability, but persistent stress can disrupt this balance. Stress may affect mood by altering the microbiota via the HPA axis. Interestingly, some NT alterations in a stressed state may be induced by the gut microbiota rather than stress itself [10]. Clinically, depressive episodes are related to HPA dysfunction. Germ-free mice exhibit a higher adrenocorticotropin and corticosterone response to restraint stress compared to regularly house-specific pathogen-free mice, suggesting a clear connection between HPA reactivity and the microbiota. The relationship between the CNS and the microbiota influences stress reactivity bidirectionally. Several studies have shown that the microbiota influences behavior and that alterations in the microbiota are linked to immunological abnormalities that impact behaviors similar to anxiety and depression [11].

The gut microbiome exerts a significant influence on the production of endocrine and metabolic substances, as well as its interactions with the host's immune and neurological systems. Particularly noteworthy is the impact of the gut microbiota on both gut and systemic inflammation, which could play a substantial role in emotional behavior changes during times of stress. Studies have revealed that individuals facing anxiety and depression often show increased plasma levels of inflammatory markers, such as tumor necrosis factor and interleukin-6 (IL-6). Recent research proposing a connection between the gut microbiome and symptoms of anxiety and depression has led to the expansion of the *microbiota-gut-brain axis* concept. This broader perspective emphasizes the intricate interplay between the brain and the gut, highlighting the gut microbiota as a vital link influencing emotional well-being [12].

Alzheimer's Disease

The primary contributors to AD are Lewy body dementia and frontal lobe dementia, which together make up approximately 70% of the cases of classic dementia [13]. AD is identified by the existence of neurofibrillary tangles and beta-amyloid (A) plaques, which result in memory impairment and cognitive deficits. In cases of gut microbial dysbiosis, the secretion of amyloid and lipopolysaccharides (LPS) occurs, disrupting both the blood-brain barrier and GI permeability. Consequently, the inflammatory signaling system is altered, triggering neuroinflammation, neuronal damage, and eventually neuronal death in AD [14]. Research indicates that disturbances in the two-way communication between the gut and the brain could play a role in neuroinflammation. The bacterial colonies within the gut microbiota release significant quantities of pro-inflammatory substances such as LPS and amyloids. These molecules can escape from the intestinal tract and accumulate in the brain, potentially contributing to neurodegeneration similar to AD and age-related inflammatory alterations.

Additionally, the gut microbiota, along with oral and respiratory bacteria, may enter the systemic circulation and access brain tissue due to a compromised blood-brain barrier. This highlights the potential role of the gut-brain axis in influencing brain health and inflammatory responses [15]. This mechanism further implicates the gut-brain axis in the pathogenesis of AD.

AD has been linked to lower levels of Fusobacteriaceae, Bifidobacterium, Actinobacteria, Firmicutes, and higher concentrations of Bacteroidetes. A neurotoxic substance called N-methylamino-L-alanine, generated by an intestinal bacterium called Cyanobacteria, disrupts the N-methyl-D-aspartate glutamate receptor and contributes to signal dysfunction in AD. Certain antibiotics and probiotics may be used as preventive measures to effectively reduce ongoing inflammation, but there is ongoing controversy regarding their impact on the microbiota. The specific composition of the microbiota is influenced by the host's genetics and nutrition, emphasizing the need for more research on the microbiota-gut-brain involvement in AD to develop novel therapeutic targets and strategies [16].

Parkinson's Disease

PD is distinguished by the buildup of misfolded alpha-synuclein protein within the dopaminergic neurons of the substantia nigra and associated circuits. This accumulation results in the manifestation of both motor and nonmotor symptoms [17]. GI dysfunction is a common symptom of PD. Various gut issues, such as infections, dysbiosis, inflammation, and dysmotility, as well as gut treatments and dietary considerations, have been linked to the onset and progression of PD, and they can also impact the response to therapy. Persistent intestinal inflammation may induce neuroinflammation and systemic inflammation, and dysfunction in the intestine can begin long before motor symptoms manifest in PD [18].

PD starts in enteric and/or nasal neurons and then spreads to the CNS via the vagal nerve and olfactory tract, respectively. It is hypothesized that α-synuclein aggregates may spread as prion-like proteins through cell-to-cell contact from the enteric nervous system to the CNS. Imbalances in the gut microbiota can overstimulate the intestinal mucosa's innate immune system, potentially activating toll-like receptor 4 and increasing oxidative stress [19]. This oxidative stress can lead to the accumulation of α-synuclein in enteric glial and enteric neurons, resulting in α-synuclein aggregation in the enteric nervous system. From there, it can travel through systemic and vagal routes to reach the brain, stimulating microglia and causing neuroinflammation and increased oxidative stress. This α-synuclein-induced microglia activation can lead to dopaminergic neurodegeneration in the nigrostriatal pathway, accelerating the development of PD.

Additionally, gut-derived microbial products may contribute to neuroinflammation and oxidative stress, potentially disrupting the blood-brain barrier in PD patients [20,21]. Gut-related features present various potential targets for the development of biomarkers and intervention methods, which should be studied in well-designed trials. Already, microbiome-directed drugs for the treatment of PD symptoms are being developed [17].

Migraines

Migraine is characterized by repeated, severe, and prolonged headaches, often accompanied by sensory hypersensitivity, visual issues, and nausea. Recent research has revealed potential causal connections between migraine and microorganisms in the GI tract. Women without migraines were found to have a significantly different microbiome profile compared to those with migraines, and there was a strong positive correlation between dysbiosis and migraine severity [22].

Increased intestinal permeability and inflammatory processes may help explain the link between GI problems and migraine headaches. When intestinal permeability increases, undigested food particles, and bacterial metabolites can enter the circulation. These bacterial endotoxins, such as LPS, may then act on the trigeminovascular system and ultimately trigger migraine-like symptoms [23]. The physiopathology of migraine is greatly influenced by a range of inflammatory and vasoactive mediators, mainly through the GI microbiota's control over the GI autonomic and immunological systems. The key pathophysiological pathways linked to migraine, including serotonergic transmission, calcitonin gene-related peptide (CGRP) activity, and cortical activation, are indirectly related to the gut flora. Considering the potential for a treatment approach based on modifying the gut microbiota or using probiotic dietary regimens, which could be cost-effective and have a good safety profile, this area presents an intriguing topic for further study [24].

Autism Spectrum Disorder

The term *ASD* refers to a range of early-appearing social communication challenges and repetitive sensory-motor behaviors. These characteristics have both strong hereditary origins and may also be influenced by other causative factors [25]. Autism is characterized by two neuropsychiatric abnormalities. The first involves impairment in the social-cognitive domain, while the second pertains to sensorimotor defects [26]. A recent study suggests a potential connection between gut flora and ASD, as many autistic children experience gut problems. GI difficulties in autistic children were observed to cause aggressive behavior, greater tantrums, and sleep disruptions, further exacerbating their behavior compared to autistic individuals without GI symptoms. The gut and the brain communicate bidirectionally, with vagal fibers playing a role in this communication. Enteroendocrine cells in the gut's epithelial barrier can detect nutrients and bacterial metabolites in the lumen and directly link the brainstem to the intestinal environment through connections with afferent vagal fibers. Mitochondrial dysfunctions can lead to intestinal dysmotility, making cells more susceptible to oxidative stress and reducing enterocyte function. The activity of caecal mitochondria may be influenced by bacterial metabolites, such as short-chain fatty acids (SCFAs) produced by Clostridia species, which can serve as energy-producing substrates through the mitochondria. Intestinal dysmotility brought on by mitochondrial abnormalities might explain constipation seen in autistic individuals [27,28].

Many individuals with ASD have a history of taking antibiotics, which can disrupt the protective microbiota while promoting the growth of anaerobic bacteria in the stomach. In ASD, common bacteria, including Clostridia, Bacteroidetes, and Desulfovibrio, may be associated with autistic behaviors and GI symptoms. These bacteria can have an impact on the gut immune system and create certain chemicals that directly contribute to the pathophysiology of autism. An approach for treating ASD may involve the use of microbiota-gut-brain axis modulators, such as helminths, probiotics, and tailored diets. Probiotics have demonstrated beneficial impacts on the gut microbiota's composition and the intestinal immune system, leading to improved intestinal barrier function, food digestion, and nutrient absorption [29].

Stroke

A stroke is a neurological condition characterized by blood vessel blockage. Blood clots impede circulation, blocking arteries, and leading to the rupture of blood vessels in the brain, resulting in bleeding. During a stroke, the arteries to the brain rupture, causing a sudden loss of oxygen and damaging brain cells [30]. Brain damage caused by a stroke can lead to the impairment or loss of cerebral function [31]. The microbiota produces or stimulates the release of various metabolites, including bile acids, indoles, SCFAs, and NTs. These metabolites enter the bloodstream and can affect the function of astrocytes, microglia, neurons, and the blood-brain barrier when they reach the brain. In several metabolic and cardiovascular conditions, such as diabetes mellitus, stroke, obesity, and hypertension, the loss of SCFA-producing bacteria has been observed. When a significant hemispheric lesion causes disrupted blood flow at the base of the middle cerebral artery for an hour, it can result in several effects, including the loss of cholinergic innervation in the ileum, heightened gut permeability, intestinal paralysis, and increased sympathetic activity. This shift from cholinergic to adrenergic transmission can cause gut inflammation. Gut dysbiosis triggered by a stroke appears to initiate a chain of events leading to poststroke infections, which is a significant factor in longer hospital stays and stroke-related mortality [32]. Understanding stroke pathogenesis and management requires investigating not only the brain but also the gut, as the gut is a significant source of inflammation and a major pathogenic element in stroke [33].

Schizophrenia

Schizophrenia has been recognized as a severe neuropsychiatric condition for over a century, but its exact cause has never been determined. Symptoms of schizophrenia include hallucinations, delusions, behavior problems, speech difficulties, and cognitive function decline. The etiology theories for schizophrenia are based on altered neurotransmission. Some studies suggest that changes in the microbiome may contribute to abnormalities in brain development, immunological control, and metabolic function associated with schizophrenia. However, establishing a direct connection between a specific microbiome and a particular mental illness can be challenging [34,35]. Research involving mice has shown that the microbiota can have an impact on cognition and the development of social behaviors, both of which are disrupted in schizophrenia. There is a link between intestinal permeability and the gut microbiota in schizophrenia, with increased bacterial translocation from the gut observed in individuals with schizophrenia. Interestingly, schizophrenia patients have been found to have higher levels of the inflammatory marker CD-14 compared to healthy individuals. Furthermore, individuals with schizophrenia exhibit lower oral microbial biodiversity, and the overall microbial composition in individuals with schizophrenia differs significantly from that of nonschizophrenic individuals. Transplanting human gut bacteria has shown promise in enhancing the treatment of schizophrenia [36,37].

Changes in the composition of intestinal bacterial populations have been associated with various conditions, including neurodevelopmental and neurological disorders such as autism, schizophrenia, PD, depression, and MS. Research is beginning to explore the mechanisms underlying the profound influence of the microbiota, including changes in energy balance, fat storage, low-grade inflammation, GI barrier function, and enhanced stress reactivity. Additionally, gut bacteria can produce neuroactive substances [19,38]. The gut microbiota can impact neurodevelopment and brain function through various routes of the gut-brain axis. The immune system plays a role in linking the gut microbiota with brain development and contributes to neuroinflammation, neuronal injury, and apoptosis. The gut microbiota influences the intestinal barrier integrity and the growth and maturity of the gut immune system [39].

In their systematic review of the use of probiotics in neurological diseases, Romijn et al. noted that further randomized controlled studies are needed before making any claims about the efficacy of probiotics for any psychological outcomes [40]. According to Ma et al., although the intricacies of the gut-brain axis remain unknown, future research must explain the proper mechanisms by which gut microorganisms' contribution leads to the regression or progression of particular clinical disorders. It serves as the foundation for advanced treatment approaches [41].

## Conclusions

Evidence is abundant concerning the gut microbiome and its interaction with the CNS. The gut-brain axis is intricately linked to various neurological disorders. Many studies have investigated these mechanisms using animal models. Currently, researchers are exploring the relevance of this intriguing pathology in human diseases. Advancements in artificial intelligence, machine learning, advanced biological techniques, metagenomics, and metatranscriptomics are currently prominent in neuroscience research. To establish this connection as pathogenic for human neurological diseases, large-scale, highly controlled clinical trials are necessary. Such trials would pave the way for interventions targeting the gut as the next-generation treatment strategy for these conditions.

## References

[1] Feigin VL, Vos T, Nichols E (2020). The global burden of neurological disorders: translating evidence into policy. Lancet Neurol.

[2] Arzani M, Jahromi SR, Ghorbani Z (2020). Gut-brain Axis and migraine headache: a comprehensive review. J Headache Pain.

[3] Morais LH, Schreiber HL 4th, Mazmanian SK (2021). The gut microbiota-brain axis in behaviour and brain disorders. Nat Rev Microbiol.

[4] Cryan JF, O’Riordan KJ, Sandhu K, Peterson V, Dinan TG (2020). The gut microbiome in neurological disorders. Lancet Neurol.

[5] Camara-Lemarroy CR, Metz LM, Yong VW (2018). Focus on the gut-brain axis: multiple sclerosis, the intestinal barrier and the microbiome. World J Gastroenterol.

[6] Malinova TS, Dijkstra CD, de Vries HE (2018). Serotonin: a mediator of the gut-brain axis in multiple sclerosis. Mult Scler.

[7] Ormstad H, Simonsen CS, Broch L, Maes DM, Anderson G, Celius EG (2020). Chronic fatigue and depression due to multiple sclerosis: Immune-inflammatory pathways, tryptophan catabolites and the gut-brain axis as possible shared pathways. Mult Scler Relat Disord.

[8] Wekerle H (2019). Secondary progressive multiple sclerosis and the gut-brain axis. Brain.

[9] Talwar D, Madaan S, Kumar S, Jaiswal A, Khanna S, Hulkoti V, Eleti MR (2021). Post Covid hypothalamic- pituitary-adrenal axis dysfunction manifesting as perinatal depression: a case series. Med Sci.

[10] Liu L, Zhu G (2018). Gut-brain axis and mood disorder. Front Psychiatry.

[11] Foster JA, McVey Neufeld KA (2013). Gut-brain axis: how the microbiome influences anxiety and depression. Trends Neurosci.

[12] Bear T, Dalziel J, Coad J, Roy N, Butts C, Gopal P (2021). The microbiome-gut-brain axis and resilience to developing anxiety or depression under stress. Microorganisms.

[13] Lekurwale V, Acharya S, Shukla S, Kumar S (2023). Neuropsychiatric manifestations of thyroid diseases. Cureus.

[14] Kesika P, Suganthy N, Sivamaruthi BS, Chaiyasut C (2021). Role of gut-brain axis, gut microbial composition, and probiotic intervention in Alzheimer's disease. Life Sci.

[15] Doifode T, Giridharan VV, Generoso JS (2021). The impact of the microbiota-gut-brain axis on Alzheimer's disease pathophysiology. Pharmacol Res.

[16] Megur A, Baltriukienė D, Bukelskienė V, Burokas A (2020). The microbiota-gut-brain axis and Alzheimer’s disease: neuroinflammation is to blame?. Nutrients.

[17] Klann EM, Dissanayake U, Gurrala A (2021). The gut-brain axis and its relation to Parkinson’s disease: a review. Front Aging Neurosci.

[18] Tan AH, Lim SY, Lang AE (2022). The microbiome-gut-brain axis in Parkinson disease - from basic research to the clinic. Nat Rev Neurol.

[19] Houser MC, Tansey MG (2017). The gut-brain axis: is intestinal inflammation a silent driver of Parkinson's disease pathogenesis?. NPJ Parkinsons Dis.

[20] Bullich C, Keshavarzian A, Garssen J, Kraneveld A, Perez-Pardo P (2019). Gut vibes in Parkinson’s disease: the microbiota-gut-brain axis. Mov Disord Clin Pract.

[21] Forsyth CB, Shannon KM, Kordower JH (2011). Increased intestinal permeability correlates with sigmoid mucosa alpha-synuclein staining and endotoxin exposure markers in early Parkinson's disease. PLoS One.

[22] Malwina M Naghibi, Richard Day (2019). The microbiome, the gut-brain axis and migraine. Gastroenterol Nurs.

[23] Dai YJ, Wang HY, Wang XJ, Kaye AD, Sun YH (2017). Potential beneficial effects of probiotics on human migraine headache: a literature review. Pain Physician.

[24] Cámara-Lemarroy CR, Rodriguez-Gutierrez R, Monreal-Robles R, Marfil-Rivera A (2016). Gastrointestinal disorders associated with migraine: a comprehensive review. World J Gastroenterol.

[25] Lord C, Elsabbagh M, Baird G, Veenstra-Vanderweele J (2018). Autism spectrum disorder. Lancet.

[26] Acharya S, Shukla S (2012). Mirror neurons: enigma of the metaphysical modular brain. J Nat Sci Biol Med.

[27] Srikantha P, Mohajeri MH (2019). The possible role of the microbiota-gut-brain-axis in autism spectrum disorder. Int J Mol Sci.

[28] Rose S, Bennuri SC, Murray KF, Buie T, Winter H, Frye RE (2017). Mitochondrial dysfunction in the gastrointestinal mucosa of children with autism: a blinded case-control study. PLoS One.

[29] Li Q, Zhou JM (2016). The microbiota-gut-brain axis and its potential therapeutic role in autism spectrum disorder. Neuroscience.

[30] Kuriakose D, Xiao Z (2020). Pathophysiology and treatment of stroke: present status and future perspectives. Int J Mol Sci.

[31] Acharya S, Shukla S, Mahajan SN, Diwan SK (2012). Localizationism to neuroplasticity---the evolution of metaphysical neuroscience. J Assoc Physicians India.

[32] Durgan DJ, Lee J, McCullough LD, Bryan RM Jr (2019). Examining the role of the microbiota-gut-brain axis in stroke. Stroke.

[33] Bonsack B, Jiang RH, Borlongan CV (2020). A gut feeling about stroke reveals gut-brain axis' active role in homeostasis and dysbiosis. J Cereb Blood Flow Metab.

[34] Nemani K, Hosseini Ghomi R, McCormick B, Fan X (2015). Schizophrenia and the gut-brain axis. Prog Neuropsychopharmacol Biol Psychiatry.

[35] Patel KR, Cherian J, Gohil K, Atkinson D (2014). Schizophrenia: overview and treatment options. P T.

[36] Kanji S, Fonseka TM, Marshe VS, Sriretnakumar V, Hahn MK, Müller DJ (2018). The microbiome-gut-brain axis: implications for schizophrenia and antipsychotic induced weight gain. Eur Arch Psychiatry Clin Neurosci.

[37] Vafadari B (2021). Stress and the role of the gut-brain axis in the pathogenesis of schizophrenia: a literature review. Int J Mol Sci.

[38] Bioque M, González-Rodríguez A, Garcia-Rizo C (2021). Targeting the microbiome-gut-brain axis for improving cognition in schizophrenia and major mood disorders: a narrative review. Prog Neuropsychopharmacol Biol Psychiatry.

[39] Yuan X, Kang Y, Zhuo C, Huang XF, Song X (2019). The gut microbiota promotes the pathogenesis of schizophrenia via multiple pathways. Biochem Biophys Res Commun.

[40] Romijn AR, Rucklidge JJ (2015). Systematic review of evidence to support the theory of psychobiotics. Nutr Rev.

[41] Ma Q, Xing C, Long W, Wang HY, Liu Q, Wang RF (2019). Impact of microbiota on central nervous system and neurological diseases: the gut-brain axis. J Neuroinflammation.

